# Descending colo-colonic intussusception secondary to signet ring cell carcinoma: A case report

**DOI:** 10.3892/ol.2014.2805

**Published:** 2014-12-17

**Authors:** KE-KANG SUN, DONG YANG, MINGQIANG GAN, XIAO-YANG WU

**Affiliations:** 1 Department of Gastrointestinal Surgery, Kunshan First People’s Hospital Affiliated to Jiangsu University, Kunshan, Jiangsu 215300, P.R. China; 2 Multiple Disciplinary Team (MDT) of Gastrointestinal Cancer, Kunshan First People’s Hospital Affiliated to Jiangsu University, Kunshan, Jiangsu 215300, P.R. China

**Keywords:** adult intussusception, signet ring cell carcinoma, descending colon cancer

## Abstract

The incidence of intussusception is low in adults, particularly in the descending colon, due to the anatomical attachment of the descending colon to the retroperitoneum. Signet ring cell histology represents ~1% of colon adenocarcinomas and is associated with young patients and a poor clinical outcome. The present study describes a case of descending colo-colonic intussusception caused by signet ring cell carcinoma in a 27-year-old male. The patient presented with a history of intermittent left upper-quadrant abdominal pain for more than six months without any evident etiology. A computed tomography scan of the abdomen revealed left-sided colo-colonic intussusception. Upon laparotomy, a left hemicolectomy was performed according to intraoperative frozen-section pathology. Post-operative pathological evaluation revealed signet ring cell carcinoma invasion of the serosa, and 31.8% (7/22) of the regional lymph nodes were positive for cancerous cells. The post-operative course was uneventful and the patient was discharged on the tenth post-operative day.

## Introduction

The majority of cases of intussusception occur in infants and children, although ~5% of affected patients are adults (1). Intussusception is the invagination of a segment of bowel into the distal adjacent bowel (2). Occurrence in the colon in adults is rare and often originates from a malignant neoplasm (3). Intussusception poses a significant challenge due to the variety of symptoms that they may present with, the diagnostic difficulties of radiological confirmation and the management of the condition.

Signet ring cell carcinoma of the colon is a rare histological subtype and accounts for 0.5–1% of all colon adenocarcinomas. The neoplasm is characterized by a specific morphological appearance of abundant intracytoplasmic mucin, which pushes the nucleus to the periphery of the cell to give a signet ring appearance (4,5). As the clinical symptoms tend to be delayed, the majority of cases of signet ring cell carcinoma are often detected at an advanced stage.

In the present study, a young male with adult intussusception of the descending colon, caused by signet ring cell carcinoma, was pre-operatively diagnosed by a computed tomography (CT) scan. Written informed consent was obtained from the patient.

## Case report

**Figure 1:**
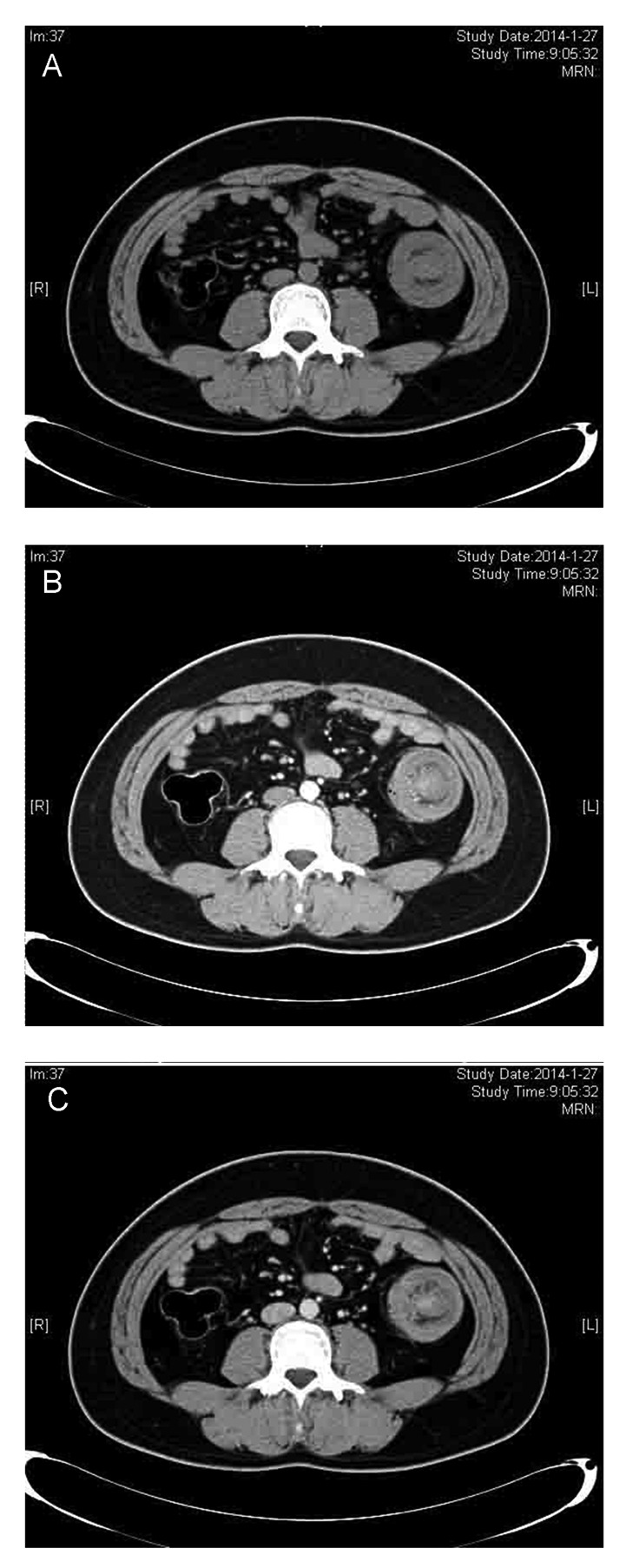
A) Unenhanced, (B) arterial phase and (C) portal venous phase computed tomography images revealing a typical target lesion associated with descending colo-colonic intussusception.

A 27-year-old male who had experienced intermittent left upper-quadrant abdominal pain without hematochezia for more than six months was admitted to the Kunshan First People’s Hospital Affiliated to Jiangsu University (Kunshan, China). The patient did not complain of any other symptoms, such as vomiting, nausea or fever, had no relevant medical or surgical history, and denied smoking and alcohol consumption. A physical examination identified left upper quadrant tenderness, but no rebound tenderness. Upon palpation, no abdominal mass was identified and laboratory test results were all within normal ranges. The chest X-ray, plain abdominal X-ray and electrocardiography findings were non-specific. A CT scan showed the concentric-like structure of the descending colon, which was typical of left colonic intussusception, but no evidence of ischemic changes in the proximal colon were revealed (Fig. 1).

**Figure 2:**
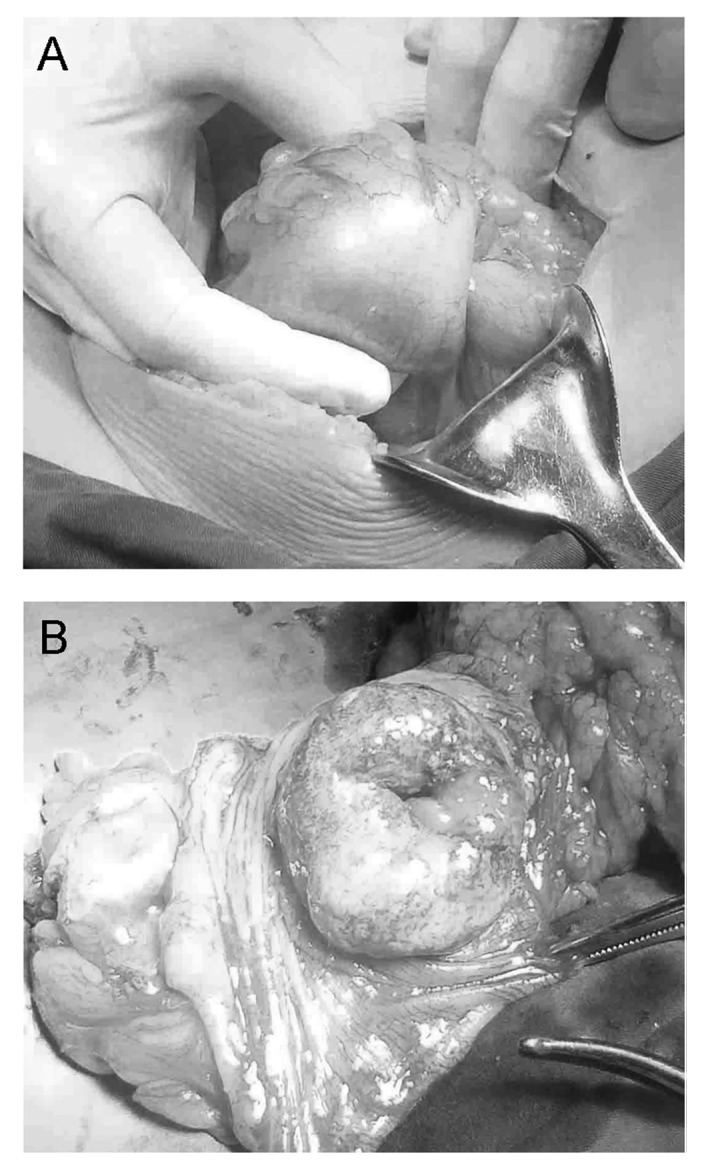
Pre-operative view of the intussusception site. (A) Surgery revealed that the tumor had caused the descending colon to intussuscept into itself. (B) The tumor *in situ*. A left hemicolectomy, which extended to the left region of the transverse colon and a section of the sigmoid colon, was performed.

Upon examination, there was no evidence of intestinal obstruction. Elective surgery with a median laparotomy was performed, which confirmed the diagnosis of intestinal intussusception caused by a left colon carcinoma. Surgery revealed that the tumor had caused the descending colon to intussuscept into itself (Fig. 2A). To prevent the neoplastic spread of the malignant tumor, manual disinvagination to save the intestinal segments was not attempted. Instead, a left hemicolectomy, which extended to the left region of the transverse colon and a section of the sigmoid colon, was performed (Fig. 2B). Intestinal continuity was restored with an end-to-side anastomosis between the transverse colon and sigmoid colon. The histology report demonstrated a signet ring cell carcinoma, which was classified as pT4aN2M0, according to the American Joint Committee on Cancer TNM classification of malignant tumors (6). The post-operative course was uneventful and the patient was discharged on the tenth post-operative day.

## Discussion

Intussusception is the telescoping of the proximal intestinal wall into the lumen of a distal intestinal segment (2,7–9). Whilst common in young patients, intussusception is rare in adults and is primarily precipitated by a malignant neoplasm. Intussusceptions have been classified into four categories according to location, as follows: i) Entero-enteric, ii) colo-colonic, iii) ileo-colonic and iv) ileo-cecal (10). Colo-colonic intussusceptions are often located in the sigmoid colon or cecum (11). Intussusceptions of the descending colon are more uncommon due to the anatomical attachment of the descending colon to the retroperitoneum (12).

The first described case of primary signet ring cell carcinoma of the colon and rectum was in 1951 (13). This particular type of neoplasm accounts for <1% of all reported adenocarcinomas and most frequently presents in young children. Furthermore, regardless of tumor location, signet ring histology is associated with a more advanced stage and higher tumor grade upon presentation, and a poorer clinical outcome compared with other subtypes of adenocarcinoma (4,14).

In the present study, a rare case of descending colo-colonic intussusception caused by a primary signet ring cell carcinoma of the colon was described. Unlike cases of childhood intussusception, no attempt should be made to reduce a colonic intussusception in adults by a barium enema; adult intussusception is often accompanied with organic diseases and, in this case, barium enema is ineffective in reduction (15,16). The reduction of intussusceptions with suspected malignancy is generally not advisable due to the possibility of bowel perforation and tumor cell dissemination. The primary treatment approach for intussusceptions is surgery (17). For lesions of the descending colon, resection of the left half of the transverse descending and sigmoid colon should be performed alongside anastomosis of the proximal transverse colon to the rectosigmoid. The performance of an anastomosis with or without proximal decompression depends upon the judgment and skill of the surgeon, and the condition of the bowel.

In the present case, one month followng surgery, the patient received six cycles of standardized chemotherapy, with each cycle lasting for four weeks, at the Department of Oncology, Kunshan First People’s Hospital Affiliated to Jiangsu University. The latest CT scan, three months after the cyclo of chemotherapy, did not show any recurrence. In a previous study, a total of 60% of colonic intussusceptions were caused by malignant neoplasms, in patients with colonic intussusceptions (18). It may be difficult to distinguish between colonic intussusceptions that harbour a benign or a malignant lesion. Therefore, we propose that in colonic intussusceptions, colonoscopy is a necessary preoperative procedure.
